# Relationship between the Health Literacy and Self-Medication Behavior of Primary Health Care Clientele in the Hail Region, Saudi Arabia: Implications for Public Health

**DOI:** 10.3390/ejihpe13060080

**Published:** 2023-06-18

**Authors:** Aidah Sanad Alqarni, Eddieson Pasay-an, Reynita Saguban, Dolores Cabansag, Ferdinand Gonzales, Sameer Alkubati, Sandro Villareal, Grace Ann Lim Lagura, Salman Amish Alshammari, Bader Emad Aljarboa, Romeo Mostoles

**Affiliations:** 1 Department of Medical-Surgical Nursing, College of Nursing, King Khalid University, Abha 62521, Saudi Arabia; 2 Maternal and Child Nursing Department, College of Nursing, University of Hail, Hail 2440, Saudi Arabia; g.lagura@uoh.edu.sa; 3 College of Nursing, University of Hail, Hail 81491, Saudi Arabia; r.saguban@uoh.edu.sa (R.S.); d.cabansag@uoh.edu.sa (D.C.); s.villareal@uoh.edu.sa (S.V.);; 4 Medical Surgical Nursing Department, College of Nursing, University of Hail, Hail 2440, Saudi Arabia; s.alkubati@uoh.edu.sa; 5 King Khalid Hospital, Ministry of Health, Hail 55421, Saudi Arabia; saalshammari@moh.gov.sa; 6 Health Cluster, Ministry of Health, Hail 55421, Saudi Arabia

**Keywords:** health literacy, self-medication, medication scale, Saudi Arabia

## Abstract

Background and aim: Because they are unaware of the potential adverse effects of medications, people frequently self-medicate as a form of self-care. This study aimed to investigate the factors associated with health literacy and the propensity to self-medicate among the primary healthcare clientele of the city of Hail, Saudi Arabia. Methods: This research employed a cross-sectional approach with the participation of 383 primary health center clientele of the Hail Region of Saudi Arabia. Participation was enacted via convenience sampling from December 2022 to February 2023. The data were collected using a self-administered questionnaire. The investigation utilized descriptive statistics as well as multiple linear regression and correlation for the data analysis. Results: Participants who were aged 30 years and above, single, had a college degree, were non-Saudi, had a white-collar occupation and received information from the internet/Google/YouTube had a significant relationship (*p* < 0.05) with health literacy. On the self-medication scale (SMS), there were significant relationships with age, marital status, educational level and occupation (*p* < 0.05). The nationality and source of information factors related to health had a positively significant effect on health literacy (*p* < 0.01), while middle age (24–29 years) had a positive effect on the self-medication scores (*p* < 0.01). There was a significant positive correlation between the health literacy screening scale (BRIEF) and the self-medication scale (SMS) scores (r = 421, *p* < 0.001). Conclusion: Age of 30 years old or above, single status, a college degree, non-Saudi status, white-collar occupation and receiving information from the internet/Google/YouTube were all significant for health literacy. There were also significant relationships with the SMS scores for age, marital status, educational level and occupation. The factors affecting health literacy were older participant age, nationality and the source of information regarding health. Conversely, among the participants, being in the middle-aged group (24–29 years) was a factor that affected their self-medication scores. There was a significant positive correlation between the health literacy screening scale (BRIEF) and the self-medication scale (SMS).

## 1. Introduction

Health literacy (HL) is a concept that is gaining popularity as a means of improving health outcomes and self-management among individuals [1,2]. It is the ability of a person to access and translate knowledge and information in order to maintain and improve health in a manner that is appropriate to the individual and system settings [3]. This involves people’s knowledge, motivation, and skills used to acquire, comprehend, assess and use health information. This is in order to make judgements and take decisions in routine activities related to healthcare, disease prevention and health promotion so as to preserve or enhance quality of life throughout the lifetime [4]. In fact, the World Health Organization [5] specifies that the cornerstone for enabling citizens to actively participate in improving their own health, engage successfully in community actions for health and pressure governments to fulfil their obligations in addressing health and health inequalities is the enhancement of health literacy in populations. As such, patients’ health outcomes are strongly predicted according to their level of health literacy, rather than their sociodemographic features [6].

Self-medication is described as the act of obtaining and ingesting medications without a doctor’s prescription, guidance or supervision during therapy. This includes taking leftover medications stored at a home location, sharing medications with family or friends or purchasing over-the-counter (OTC) medications [6]. Due to the high prevalence of diseases and the lack of medical services, poor countries are seeing an increase in the trend of self-medication [7], and negative repercussions will result in detrimental health impacts if self-medication decisions are not supported with sufficient and credible medical information [8]. According to the World Health Organization (WHO), ineffective prescribing procedures, such as improper dosing, dropped treatment sessions and careless drug use, have all aided in the development and spread of antibiotic resistance [9]. A reduction in inappropriate self-medication can be achieved by raising public health literacy levels [8].

Some research has linked poor health literacy to worse medication adherence [10,11], whereas other studies have found no association with, or even higher medication adherence among, those with poor health literacy [12,13,14,15]. The need to identify factors influencing the change in behavior required to achieve healthy behavior is necessary given the rising prevalence of self-medication in the community and the direct involvement of individuals in the selection and use of medications. This will enable people to live long, relatively healthy, active lives [16]. Many factors, including education level, social and familial pressures, drug accessibility and media exposure can influence a person’s decision to self-medicate [17]. The decision to self-medicate may also be influenced by a person’s experience with the severity and length of their condition [18]. The issue of whether health literacy has an amplifying or lowering influence on the relationship between beliefs and adherence has been raised, despite the fact that health literacy is vital for medication adherence and the association between health literacy and medication adherence is still unclear [19]. Despite the fact that health literacy practices are increasingly promoted in public health literature, there are not many studies that examine the links between health literacy and self-medication [8].

This study is significant and very timely, as it provides further understanding of how health literacy plays a role in helping individuals to self-medicate or adhere to their medications. In turn, this study boosts the degree of public health literacy as one of the most effective factors in lowering the practice of self-medication, which can reduce the harms associated with it considerably. Data derived from this study can offer crucial preliminary information to policymakers and health professionals for improving health literacy and encouraging public understanding and literacy of self-medication and can serve as a foundation for future research. In light of the fact that health literacy can offer useful guidelines for creating interventions aimed towards enhancing patients’ knowledge and abilities to discern the effects of self-medication, it is therefore hypothesized that health literacy impacts the propensity to self-medicate. As such, this study sought to answer the following research questions: What is the relationship between the participants’ characteristics, health literacy, and self-medication? What are the factors affecting health literacy and self-medication scale scores? What is the relationship between health literacy and self-medication? This study aimed to investigate the factors associated with health literacy and the propensity to self-medicate among the primary healthcare clientele of the city of Hail, Saudi Arabia.

## 2. Materials and Methods

### 2.1. Design

This study employed a cross-sectional approach to investigate the factors associated with health literacy and the propensity to self-medicate among the primary healthcare clientele of the city of Hail, Saudi Arabia.

### 2.2. Setting/Sampling

The study took place in the Hail Region, specifically in the 15 primary healthcare centers of the city of Hail. The RAOSOFT calculator was used to calculate the number of participants that were required based on a metro area population of 413,000 in 2022 (https://www.macrotrends.net; accessed on 20 December 2022). Convenience sampling was utilized, yielding 384 participants. The participants in this study were clients who were visiting the primary healthcare centers for check-ups. The eligibility criteria included: (a) age of at least 18 years old or above; (b) ability to read and comprehend English; and (c) willingness to participate.

### 2.3. Data Collection

Data collection started after ethical clearance was approved. Informed consent was explained to the participants, as were the aim of the study, the extent of their participation, and their rights as participants. The participants were given a paper-based questionnaire during their visits to the primary healthcare centers. They were asked to read the informed consent form before proceeding to answer the questionnaire. A minimum of 15 min was given for the participants to answer the questionnaire; they could extend this time based on their pacing. Data collection started in January and concluded in March 2023.

### 2.4. Instruments

In addition to collecting the demographic characteristics of the participants, two questionnaires were used in this study. These included the BRIEF Health Literacy Screening Tool, a validated 4-item screening questionnaire developed by Haun et al. [20] that evaluates the patient’s capacity for understanding health information. It included questions such as: (1) ‘How often do you get someone to study hospital documents with you?’ (2) ‘How comfortable are you completing medical forms on your own?’ (3) ‘How frequently do you find it difficult to grasp written information regarding your medical condition?’ (4) ‘How confident are you filling out medical forms by yourself?’ Participants responded to each question on a 5-point scale. The scores ranged from 2–20, with 2–12 = inadequate HL, 13–16 = marginal HL, and 17–20 = adequate HL. The BRIEF requires less time for administration and scoring than the original questionnaire, and it provides a benefit over the original questionnaire, which entails an evaluation of patients’ literacy abilities, in that it is less likely to cause patient embarrassment.

The second questionnaire assessed resistance to self-medication, comfort with self-medication, and views about allowing events to play out naturally; it was adapted from James and French [21]. It had nine items that were answered on a 5-point Likert scale ranging from 1 = Rarely, 2 = Not that often, 3 = Sometimes, 4 = Often and 5 = Very often. Negative statements such as items 7 and 8 were reversed. A high score indicated a greater propensity to self-medicate.

### 2.5. Ethical Consideration

This research was conducted with the approval of the Institutional Review Board of the University of Hail (H-2022-001). The participants were assured that all data gathered would be treated with the utmost confidentiality.

### 2.6. Statistical Analysis

The collected data were analyzed using the SPSS version 26: Frequency and percentage were used to represent the descriptive variables. Multiple regression was used to predict the factors associated with health literacy and self-medication. The bivariate r was used to determine the association between health literacy and the propensity to self-medicate.

## 3. Results

The demographic and occupational characteristics of the general population (N = 384) are illustrated in Table 1. More than one-third of the participants were over 30 years old and single (41.1% and 39.8%, respectively). Regarding the educational level, approximately one-third either had completed college or were still engaged in their studies (32.6% and 35.4%, respectively), while the fewest of them were in elementary school. Non-Saudi nationality formed more than half of the participants (52.1%). Roughly two-thirds (65.9%) had a blue-collar occupation, and more than half (56.3%) received information on health from the internet, Google or YouTube.

Table 2 shows significant relationships between all the studied variables and the scores of the health literacy scale (BRIEF). Participants who were aged 30 years old or above, single, had a college degree, were non-Saudi, had white-collar occupation and received information from the internet/Google/YouTube were significant (*p* < 0.05) according to BRIEF. Regarding the self-medication scale (SMS), there were significant relationships between age, marital status, educational level and occupation. In comparison, there was no significant relationship between the scores of the SMS and nationality or the source of information (*p* ˃ 0.05).

The multiple linear regression of the factors affecting the health literacy and self-medication scale scores are shown in Table 3. Older age of the participants, nationality and source of information on health had positively significant effects on health literacy, while marital status had a negatively significant effect on it (*p* < 0.01). In comparison, for the participants, being in the middle-aged group (24–29 years) had a positive effect on the self-medication scores (*p* < 0.01). By contrast, nationality and the participants having completed secondary education had negative effects on the self-medication scores (*p* < 0.01).

**Figure 1:**
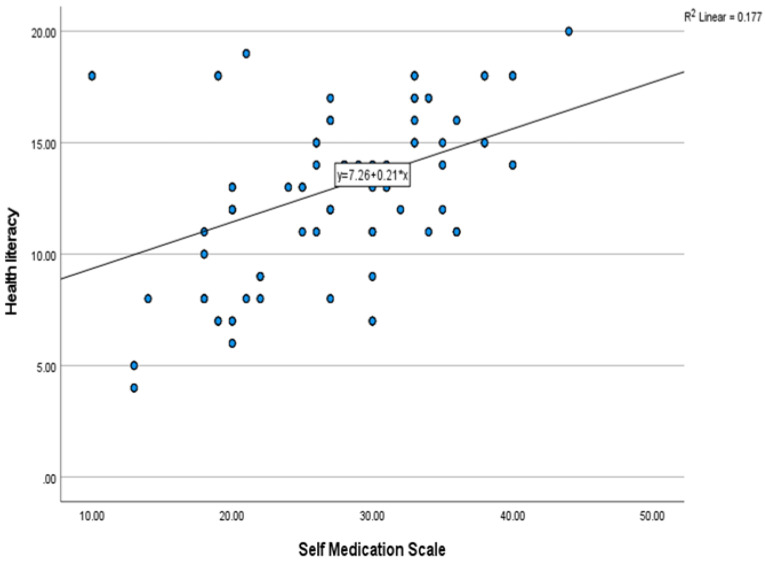
Pearson’s correlation between health literacy and self-medication scale scores.

Figure 1 illustrates a significant positive correlation between the health literacy screening scale (BRIEF) and the self-medication scale (SMS) scores (r = 421, *p* < 0.001).

## 4. Discussion

This study investigated factors associated with health literacy and the propensity to self-medicate among the primary healthcare clientele of the city of Hail, Saudi Arabia. Study participants who were aged 30 years old or above, single, had a college degree, were non-Saudi, had a white-collar occupation and received information from the internet/Google/YouTube scored significantly higher than others did on the health literacy scale. This means that certain demographic factors have an impact on the health literacy scale. When comparing people of all ages in Saudi Arabia and abroad, researchers discovered that those aged 30 or older had the highest health literacy [22,23]. This implies that growing older and gaining more life experience can enhance one’s health literacy. Therefore, it is reasonable to conclude that young adults (those aged 29 and under) should be among the primary foci of future health literacy initiatives.

A higher level of health literacy was also seen among those with a college degree. This indicates that, as with the impacts of age, health literacy can be improved through a combination of formal education, the right training, experience in one’s chosen area and wisdom gained from the struggles and successes of life. Comparable conclusions were reached from investigations in Saudi Arabia and elsewhere [22,23,24]. One of the strongest indicators of a patient’s ability to understand and use health information is their level of formal education [25]. Poorly educated individuals struggle to grasp and evaluate health information, resulting in low health literacy [26]; hence, they are unable to express their needs to healthcare professionals. Accordingly, those who complete higher education at the university level are typically employed in white-collar positions, as their formal education and skills make them indispensable to their employers. This conceivably explains why those with white-collar occupations scored higher in the literacy scale than those with blue-collar jobs. Pei and colleagues [27] found that, compared to their white-collar peers, blue-collar workers were less likely to seek medical care when they were ill. According to a Japanese survey, white-collar workers in large corporations in Japan enjoy higher salaries and greater job security due to lifelong employment [28]. Naturally, smaller businesses devote less resources to health promotion initiatives in the workplace, while employees at larger firms have greater access to paid time off and are more likely to see a doctor [27]. Educative interventions aiming to improve health literacy in low-literacy populations should, therefore, be planned and implemented with the highest priority.

Regarding civil status, those who were single scored higher, which indicates that unmarried individuals might be less likely to partake in unhealthy activities, eat poorly and neglect their health. They also outperformed the norm in terms of both the frequency of doctor visits [29] and the consistency with which they implemented their doctors’ recommendations. Numerous studies involving a wide range of participants found similar results [29,30]; however, past investigations concluded otherwise [31,32,33]. This disparity may emerge because it is possible that the lack of social and familial assistance for health issues is related to the fact that these individuals tend to live alone. Moreover, married people have more responsibilities and, hence, less time to devote to health-related concerns. No significant differences in marital status were reported by Azlan and colleagues [34]; however, this could be due to the young age of the sample. It is noteworthy that separated and divorced individuals may have trouble attending health education classes owing to emotional and mental health concerns [35]. Health literacy programs must take the unique circumstances of these individuals into consideration.

In the present study, non-Saudi individuals scored significantly higher than their Saudi counterparts, indicating that both linguistic and ethnic factors contribute to the discrepancy in perspectives. Low health literacy has been observed to be more prevalent in communities whose members do not speak English as their first language [36]. Another possible explanation for the disparity in health literacy is that non-Saudis are mandated to have health insurance, whilst Saudis are routinely provided free public healthcare [37]. Since Saudis are employed by the government, they normally have free access to public healthcare. In comparison, most non-Saudis work in the private sector and are consequently required to have health insurance, thus making them more health-literate. If the healthcare system in the Kingdom of Saudi Arabia is to realize its full potential in terms of increasing public health, its citizens must be able to easily find the services they need, understand them and put them to good use.

Since most health services in the twenty-first century can be accessed via the internet, people living in the digital age have a better appreciation of health management, and they also have easier access to these resources [38]. The results of this study showed that people who obtained their information from online sources, such the internet, Google and YouTube, outperformed those who relied on official releases from the Ministry of Health—specifically, those individuals who are well-versed in the use of many forms of media and technology and who make it a habit to use them in their pursuit of health information and care [39,40]. Consequently, the ability to comprehend and use one’s own media literacy is crucial for good health literacy [41]. Healthcare professionals can promote health resilience and disease prevention by creating techniques for obtaining self-care knowledge from a variety of sources, in addition to helping others to understand and use that information.

Regarding the resistance to self-medication scale (SMS), there were significant relationships of the total scores of the SMS with age, marital status, educational level and occupation. This means that there are effects on resistance to self-medication that can be attributed to demographic variables. According to a systematic review by Bert and associates [42], some regions were found to have a high prevalence of cases of children administering their own medications, whereas other studies identified greater prevalence in younger and middle-aged groups [43,44]. As per the research of Ahmed and Sulaiman [45], 79% of these instances included people aged 21 to 50. There were no statistically significant associations between age and self-medication in the other investigations [46]. The availability of medications for over-the-counter purchase may contribute to the observed disparities between regions [47,48].

As specified in the findings of Jafari and colleagues [49], the rate of self-medication was much greater among the single respondents than their counterparts; however, no correlation was found between the use of self-medication and marital status [50]. This disparity could be explained by the fact that single respondents are more likely to experience health problems and resort to self-medication than their married counterparts [49], and spouses may supervise prescription use. A number of studies have examined the relationship between having a moderate or high degree of education and a person’s ability to resist the urge to self-medicate. It is noteworthy that persons with higher education levels, such as a high school diploma or college degree, had a far lower likelihood of administering their own medications [51,52,53].

Regarding occupation, based on the results of the study conducted by Chautrakarn and partners [54], the incidence of self-medication among healthcare professionals was close to 88%, which is on a par with the prevalence among the non-healthcare-provider group. Knowledge of diagnosis and treatment modalities was found to be a greater motivator for self-medication among healthcare professionals [55]. These people may be more likely to self-medicate rather than see a doctor due to a lack of time, a heavy patient load and ready access to pharmaceuticals [56]. It is also noteworthy that health insurance is available for several occupations, and its presence has been linked to a decrease in the likelihood of self-medication [57].

In this study, older age among the participants, alongside nationality and the source of information on health, had a positively significant effect on health literacy, which means that a person’s health literacy improves naturally as they age. Because of their greater experience with the healthcare system and their more established relationships with their doctors, older people may be better equipped to handle these challenges. This finding supports those of other investigations [58,59], differing from what was observed in other studies [32,60]. Previously, in examining contradictory findings about the relationship between age and health literacy, van der Heide et al. [61] concluded that age was related to decreased health literacy in various health literacy domains.

As previously mentioned, regarding nationality, because of the language disparity, non-Saudis scored significantly higher than their Saudi counterparts on the health literacy scale. While these nationalities share a cultural milieu, they are distinct in numerous ways, including their religious and philosophical outlooks and their approaches to life. Research conducted by Abdel-Latif and Saad [62] showed that Saudi citizens may have trouble in comprehending and applying health resources written in English. The use of technical terms and medical jargon in health-related writing can be a major impediment to comprehension. Nearly half of the Saudi population are affected by low health literacy [63]. It is worth noting that the residents of the Hail region are statistically far less likely to have poor health literacy [64]. The success of any healthcare system depends on patients’ ability to read and understand their health data, written recommendations and medication orders. Certainly, a healthcare system can implement health literacy standards to make it easier for its patients to access and understand health information.

The same outcome can be established regarding sources of information. One’s level of health literacy increases in proportion to the amount of knowledge obtained from a variety of sources. This result substantiates those of other studies [31,64]. Methods of disseminating health information through various media are also crucial. The data should be easily understandable and practically applicable [65]. The health benefits of social media highlight the growing importance of this medium in people’s daily lives [66,67]. Nonetheless, as stated in a study of Zhang and partners [68], investigations are warranted on the veracity and security of health information found on social media.

Among the participants, being in the middle-aged range (24–29 years) had a positive effect on the self-medication scores, indicating that self-medication is more prevalent among responders as they approach middle age. This current finding is congruent with those of studies that reported on the incidence of self-medication among middle-aged individuals in China, Egypt, Lebanon, Thailand, Syria and Vietnam [54,69,70,71,72,73,74]. Although the prevalence of middle-aged adults who engage in self-medication varies between those studies, it does not fall below 67%. Recall times, healthcare facilities, the economy and social and cultural variables could all play a role in this difference. Programs aimed towards educating middle-aged persons about medication safety are strongly encouraged.

Concerning nationality, the Saudis practiced more self-medication than their non-Saudi counterparts. This is in line with a study conducted by Al-Ghamdi and associates [75], which concluded that these data may be an indication of cultural differences, because the purchasing power of Saudi Arabians is likely to be greater than that of their counterparts living in other countries, as Saudi Arabia is a more developed country with a more robust economy. Health regulators in Saudi Arabia may find this information helpful as they work to inform the general public about self-medication in an effort to reduce the risk of undesirable drug reactions and drug–drug interactions, especially among at-risk populations.

In the present study, there was a significant positive correlation between the health literacy screening scale (BRIEF) and the self-medication scale (SMS) scores, which means that cognitive abilities [76], including those required for the effective and efficient incorporation of health information, guidance, understanding, problem solving and judgment [77], are enhanced by health literacy and hence equip people to make more nuanced judgments about self-medication by allowing them to weigh the pros and cons of certain courses of action in relation to their health. Previous studies reported that the causes of self-medication may have been due to poor health literacy [14]. Consequently, there is a dire need to curb this social phenomenon, as its effects have far-reaching repercussions. The findings of this investigation corroborate those of other studies showing that health literacy correlates favorably with drug literacy and responsible drug use [78,79,80]. Conversely, the findings of the current study do not correspond to those of other studies, which revealed that patients who self-medicate also demonstrated greater levels of health literacy [81]. The discrepancy could be due to other variables, such as poverty, the fact that the participants in Mousaeipour et al.’s [82] study were women and legislation governing the trade and use of drugs. Self-medication practices can develop in different countries with varying patterns, given that most human behaviors are influenced by ideas, ideologies and conventions depending on the cultural and social environment of the area [83]. Therefore, to draw meaningful conclusions, it is important to conduct such studies among various demographics.

### Study Limitations

The authors acknowledge the limitations of this study, which could be addressed in subsequent research. The use of convenience sampling, the high percentage of participants who were non-Saudi and the fact that the sample were very young mean that the data are unlikely to be representative of the general population, which affects the validity of the data and limits the capacity to extrapolate the findings from this sample to the relevant population. Therefore, this problem could be resolved by employing random sampling in a larger context to include more Saudi citizens of varying ages in a replication study. Moreover, because the questionnaire was written in English, patients who had difficulties with this language were excluded. Future research should therefore examine these issues, since they directly relate to the educational demands of these patients.

## 5. Conclusions

The scores of primary healthcare clients who were aged 30 years old or above, single, had a college degree, were non-Saudi, had a white-collar occupation and received information from the internet/Google/YouTube were all significant with respect to health literacy. There were also significant relationships of the SMS scores with age, marital status, educational level and occupation.

The age of the participants, their nationality and sources of health information all had impacts on health literacy in primary healthcare settings of the Hail region. Conversely, among the participants, being in the middle-aged group (24–29 years) was a factor that affected self-medication scores. There was a significant positive correlation between the health literacy screening scale (BRIEF) and the self-medication scale (SMS). On the local scale, health literacy can be improved by providing primary healthcare clients in the Hail region with information that is specifically tailored to their needs and by boosting public and healthcare professional awareness of the advantages of rational drug use through social media. Healthcare practitioners with expertise in medication safety can play an important role in promoting appropriate information and the importance of avoiding self-medication. More research is needed to determine how health literacy can be applied to groups as a whole to effect cultural improvements. Furthermore, empirical research needs to be strengthened on the link between self-medication, attitudes, behavior and health outcomes, serving as a reference for improving health literacy and raising the health standard of Saudi citizens.

### Implications for Public Health

The findings that were observed from the results of our study show the necessity of including the topics of self-medication and health literacy in primary healthcare education. Accordingly, the basic goal of public health building is the implementation of policies and programs that increase health literacy on the national scale [63]. A number of interventions, including public health campaigns, strict legislation on the distribution of drugs from dispensaries, and improvements in both the performance and availability of healthcare, may be necessary in order to alter people’s health-seeking behavior and safeguard them from the dangers of self-medicating. Because a low health literacy level constitutes one of the most significant problems for both the formulation of health policies and promoting healthy behaviors, it is advised that efforts be made to acquire these skills in conjunction with initiatives that promote public health. This should be accomplished by the utilization of the capabilities of cultural, educational and media entities, as well as the organizations that fall under the supervision of the Ministry of Health.

## Figures and Tables

**Table 1:** Demographic characteristics of the participants = N = 384.

Characteristics		n	(%)
Age			
	18–23 years old	113	(29.4)
	24–29 years old	113	(29.4)
	30 years old or above	158	(41.1)
Marital Status			
	Single	153	(39.8)
	Married	137	(35.7)
	Separated/divorced	94	(24.5)
Educational Level			
	Completed college	125	(32.6)
	Did not complete college	136	(35.4)
	Completed secondary	84	(21.9)
	Elementary	39	(10.2)
Nationality			
	Saudi	184	(47.9)
	Non-Saudi	200	(52.1)
Occupation			
	Blue-collar occupation	253	(65.9)
	White-collar occupation	131	(34.1)
Source of Information on Health			
	Announcement from the Ministry of Health	168	(43.8)
	Internet/Google/YouTube	216	(56.3)

**Table 2:** Relationship between participant characteristics and the BRIEF and SMS scores.

Age	Group	BRIEF			SMS		
		Mean Rank	Median (IQR)	*p*-Value	Mean Rank	Median (IQR)	*p*-Value
	18–23 years old	168.95	12 (11–15)	<0.001	147.65	25 (20–27)	<0.001
	24–29 years old	180.28	12 (11–15)		215.09	30 (26–34)	
	30 years old and above	218.08	14 (11–17)		208.42	28 (22–34)	
Marital Status							
	Single	210.49	14 (12–16)	0.033	165.25	26 (20–29)	<0.001
	Married	182.59	12 (9–17)		215.34	30 (22–35)	
	Separated/divorced	177.66	13 (11–14)		203.56	28 (21–33)	
Educational Level							
	Completed college	209.52	15 (11–18)	<0.001	198.28	30 (26–33)	0.001
	Did not complete college	153.42	12(11–14)		164.22	27 (20–33)	
	Completed secondary	150.37	13 (8–14)		149.59	25 (20–28)	
	Elementary		14 (11–15)			29 (21–33)	
Nationality							
	Saudi	180.12	13 (8–15)	0.035	196.84	27 (21–33)	0.461
	Non-Saudi	203.89	13 (11–16)		188.51	28 (20–33)	
Occupation							
	Blue-collar occupation	173.79	13 (10–15)	<0.001	181.66	27 (20–33)	0.008
	White-collar occupation	228.63	14 (11–18)		213.44	30 (24–33)	
Source of Information on Health							
	Announcement from the Ministry of Health	173.14	12 (11–14)	0.002	195.90	27 (20–35)	0.596
	Internet/Google/YouTube	207.56	14 (11–17)		189.86	27 (21–33)	

**Table 3:** Factors affecting health literacy and self-medication scale scores.

	Unstandardized Coefficients		Beta	t	Sig.	95.0% Confidence Interval for B	
	B	Std. Error				Lower Bound	Upper Bound
(Constant)	8.902	1.582		5.629	0.000	5.792	12.012
24–29 years old	0.234	0.572	0.029	0.409	0.683	−0.891	1.358
30 years old or above	2.828	0.614	0.375	4.608	0.001	1.621	4.035
Married	−2.131	0.475	−0.275	−4.490	0.001	−3.065	−1.198
Divorced	−2.791	0.657	−0.323	−4.246	0.001	−4.083	−1.499
Did not complete college	−1.097	0.717	−0.141	−1.531	0.127	−2.506	0.312
Completed secondary school	−1.236	0.779	−0.138	−1.587	0.113	−2.767	0.295
Elementary	−0.861	0.683	−0.070	−1.261	0.208	−2.204	0.482
Nationality	1.202	0.444	0.162	2.707	0.007	0.329	2.075
Occupation	0.873	0.681	0.112	1.282	0.200	−0.466	2.212
Source of information on health	1.294	0.363	0.173	3.563	0.001	0.580	2.009
Self-Medication			beta	t	Sig.	95.0% Confidence Interval for B	
	B	Std. Error				Lower Bound	Upper Bound
(Constant)	35.304	3.383		10.436	<0.001	28.652	41.957
24–29 years old	4.863	1.223	0.297	3.977	0.001	2.459	7.268
30 years old or above	2.425	1.313	0.160	1.847	0.066	−0.157	5.006
Married	0.292	1.015	0.019	0.288	0.774	−1.704	2.289
Divorced	2.240	1.406	0.129	1.593	0.112	−0.525	5.004
Did not complete college	−2.948	1.533	−0.189	−1.923	0.055	−5.963	0.066
Completed secondary school	−5.555	1.666	−0.308	−3.335	<0.001	−8.830	−2.280
Elementary	−1.782	1.461	−0.072	−1.219	0.223	−4.655	1.091
Nationality	−4.161	0.950	−0.278	−4.381	<0.001	−6.029	−2.293
Occupation	−1.670	1.457	−0.106	−1.146	0.252	−4.534	1.195
Source of information on health	−0.050	0.777	−0.003	−0.064	0.949	−1.578	1.478

## Data Availability

The data presented in this study are available on request from the corresponding author. The data are not publicly available due to privacy concerns.
